# Trends in prevalence of unmet need for family planning in India: patterns of change across 36 States and Union Territories, 1993–2021

**DOI:** 10.1186/s12978-024-01781-6

**Published:** 2024-04-09

**Authors:** Kirtana Devaraj, Jewel Gausman, Raman Mishra, Akhil Kumar, Rockli Kim, S. V. Subramanian

**Affiliations:** 1 Department of Chemical Engineering and Applied Chemistry, University of Toronto, Toronto, Canada; 2 Department of Global Health and Population, Harvard T. H. Chan School of Public Health, Boston, MA USA; 3 Maternal and Child Health Nursing Department, School of Nursing, University of Jordan, Amman, Jordan; 4 Interdisciplinary Program in Precision Public Health, Department of Public Health Sciences, Graduate School of Korea University, Seoul, Republic of Korea; 5 Faculty of Arts and Science, University of Toronto, Toronto, Canada; 6 Division of Health Policy and Management, College of Health Science, Korea University, Seoul, Republic of Korea; 7 Harvard Center for Population and Development Studies, 9 Bow Street, Cambridge, MA USA; 8 Department of Social and Behavioral Sciences, Harvard T. H. Chan School of Public Health, Boston, MA USA

**Keywords:** Family planning, Unmet need, NFHS, India, States, Union Territories

## Abstract

**Background:**

Eliminating unmet need for family planning by 2030 is a global priority for ensuring healthy lives and promoting well-being for all at all ages. We estimate the sub-national trends in prevalence of unmet need for family planning over 30 years in India and study differences based on socio-economic and demographic factors.

**Methods:**

We used data from five National Family Health Surveys (NFHS) conducted between 1993 to 2021 for the 36 states/Union Territories (UTs) of India. The study population included women of ages 15–49 years who were married or in a union at the time of the survey. The outcome was unmet need for family planning which captures the prevalence of fecund and sexually active women not using contraception, who want to delay or limit childbearing. We calculated the standardized absolute change to estimate the change in prevalence on an annual basis across all states/UTs. We examined the patterning of prevalence of across demographic and socioeconomic characteristics and estimated the headcount of women with unmet need in 2021.

**Results:**

The prevalence of unmet need in India decreased from 20·6% (95% CI: 20·1– 21·2%) in 1993, to 9·4% (95% CI: 9·3–9·6%) in 2021. Median unmet need prevalence across states/UTs decreased from 17·80% in 1993 to 8·95% in 2021. The north-eastern states of Meghalaya (26·9%, 95% CI: 25·3–28·6%) and Mizoram (18·9%, 95% CI: 17·2–20·6%), followed by the northern states of Bihar (13·6%, 95% CI: 13·1–14·1%) and Uttar Pradesh (12·9%, 95% CI: 12·5–13·2%), had the highest unmet need prevalence in 2021. As of 2021, the estimated number of women with an unmet need for family planning was 24,194,428. Uttar Pradesh, Bihar, Maharashtra, and West Bengal accounted for half of this headcount. Women of ages 15–19 and those belonging the poorest wealth quintile had a relatively high prevalence of unmet need in 2021.

**Conclusions:**

The existing initiatives under the National Family Planning Programme should be strengthened, and new policies should be developed with a focus on states/UTs with high prevalence, to ensure unmet need for family planning is eliminated by 2030.

**Supplementary Information:**

The online version contains supplementary material available at 10.1186/s12978-024-01781-6.

## Abstract (plain language summary)

This study looked at the trends in unmet need for family planning in India, which is defined as the percentage of women of reproductive age who want to delay or limit childbearing but are not using any contraceptive method. A public dataset was used to analyze national and sub-national trends from 1993 to 2021. It was determined that although the percentage prevalence of unmet need decreased in the last 30 years, there were still a substantial number of women with unmet need in 2021. More than half of these women were in Uttar Pradesh, Bihar, Maharashtra, and West Bengal. Furthermore, it was found that percentage prevalence of unmet need was relatively higher amongst younger women and those belonging to poorer households in 2021. Initiatives and policies aimed at reducing unmet need for family planning should be implemented while considering geographic, socioeconomic, and demographic differences.

**Supplementary Information:**

The online version contains supplementary material available at 10.1186/s12978-024-01781-6.

## Background

Unmet need for family planning refers to the percentage of fecund and sexually active women who are not using a contraceptive method, but report not wanting another child or wanting to delay their next pregnancy [1]. Globally, it has been estimated that 164 million women had an unmet need for family planning in 2021 [2].

The Sustainable Development Goal (SDG) of good health and well-being aims to ensure healthy lives and promote well-being for all at all ages [3]. Various indicators are used to quantify progress made towards achieving this goal. SDG indicator 3·7·1 quantifies the proportion of women of ages 15–49 years who have their need for family planning satisfied with modern methods, also referred to as the demand satisfied by modern methods [4]. The components of this indicator are contraceptive prevalence and unmet need for family planning [5]. There are two types of unmet need: spacing—women who do not want another child for at least 2 years or do not know when they want another child, and limiting—women who do not want another child at all [1].

The International Conference on Population and Development (ICPD) recognizes reproductive health and the empowerment of women as pillars of sustainable development. It was established in 1994 with representatives of 179 governments and a 25 year review was held in 2019 (ICPD + 25), which aimed to renew momentum to achieve the ICPD Programme of Action and the SDGs by 2030. In ICPD + 25, zero unmet need for family planning information and services by 2030 was identified as a target for achieving universal access to sexual and reproductive health and rights [6]. The conference also recognized that reproductive rights and the reproductive health of women can only be realized with adequate access to family planning services, including education and contraception. These services empower women to make well-informed choices, leading to improved reproductive health outcomes [7].

Studies have projected that unmet need for family planning worldwide will be greater than 10% by 2030 [8]. As the most populous country in the world with a population of 1·4 billion, India has a significant role in ensuring the success of the world in eliminating unmet need [9]. This is contingent upon India’s progress in identifying high-risk populations for unmet need and implementing measures tailored towards the needs of various sections of the population. Globally, demographic and socioeconomic characteristics such as age, place of residence, and education level have been shown as determinants of unmet need in studies focusing on specific developing nations [10, 11]. Similar challenges have been identified in India, with a particular emphasis on subnational geographic variation [12]. India is a federation of 28 states and 8 UTs [13, 14]. States are composed of geographical units known as districts and have their own legislatures, and fall in the jurisdiction of state governments [15]. UTs fall under the governance of the central government and may have their own legislatures. There are substantial differences in indicators related to population health and well-being across states and UTs [16, 17].

In this study, we present a comprehensive and systematic description of the trends in prevalence of unmet need for family planning among married/in union women of ages 15–49 years at the time of the survey, in India and its 36 States/Union Territories (UTs) between 1993 and 2021. From a policy perspective, understanding the geographical distribution of unmet need could aid in forming policies targeted towards each state/UT. In addition to prevalence, knowing the absolute burden *i.e.,* the current headcount of women with unmet need for family planning is important, as the headcount may not necessarily follow the same pattern as prevalence. The headcount estimate may help ensure that services are provided at adequate capacity in all states/UTs. Therefore, we estimated the absolute headcount burden of women with unmet need for family planning for 2021 for India and its states/UTs. We evaluated the patterning of unmet need across basic demographic and socioeconomic characteristics in 2021. Last, we assessed which states/UTs are on track to achieve zero unmet need by 2030.

## Methods

### Data

This study used data from five waves of the National Family Health Survey (NFHS), conducted in 1992–93, 1998–99, 2005–06, 2015–16 and 2019–21 [18–22], hereafter identified with the end year of each survey. These surveys covered all states and UTs in India and are publicly accessible from the website of the Demographic and Health Surveys [23]. All surveys employ a multi-stage stratified cluster-sampling design and use the latest available Census of India at the time survey. In each survey, Primary Sampling Units (PSUs), known as villages in rural areas, and wards in urban areas were selected. Households were then randomly selected from each PSU. Microdata available in each survey was used in this study.

### Sample Population

The sample population constitutes of women aged 15 to 49 years who are married or in a union, at the time of the survey. The sample population has been restricted to the currently married/in union population based on the revised unmet need definition proposed by Bradley et al. [24]. The first three rounds of the NFHS had the response option of “currently married” for the question *“What is your current marital status”* that is relevant to this study. The last two rounds had the option of “currently in union/living with a man”. In this study, the “currently married” and “currently in union/living with a man” responses have been considered as one and represented as the currently married/in union group. Women who did not answer all the required questions to determine unmet need were excluded from this analysis. The final study sample population is presented in Table 1.

**Table 1:** Study Sample Size from the five National Family Health Surveys, 1993–2021

**Survey round (year)**	**Sample size based on inclusion criteria (n)**	**Non-responses on unmet need related questions (n)**	**Final study sample size (n)**
NFHS-1 (1992–93)	84,289	1,183	83,106
NFHS-2 (1998–99)	84,862	23	84,839
NFHS-3 (2005–06)	87,925	121	87,804
NFHS-4 (2015–16)	499,627	4	499,623
NFHS-5 (2019–21)	512,408	76	512,332
**All Waves**	**1,269,111**	**1,407**	**1,267,704**

NFHS-1, NFHS-2, and NFHS-3 had the following survey response option relevant to the study for a question on marital status: currently married. NFHS-4, and NFHS-5 had the following survey response: currently in union/living with a man. For the purposes of this study, these responses have been combined and represented as one group: currently married/in union

### Outcome

Women were defined as having unmet need for spacing or limiting based on the definition presented by Bradley et al. [24]. Women with unmet need for spacing and limiting were distinguished based on questions related to desire of current pregnancy (if pregnant), desire of last pregnancy, and ideal timing of next pregnancy if desired. The full technical details on the calculation of unmet need for spacing and limiting are published elsewhere [24].

Women with unmet need for spacing were defined as: pregnant women who wanted current pregnancy later, postpartum amenorrheic women (for less than 24 months) not using contraception who wanted last birth later, or women who want children after 2 + years, or are undecided about the timing, or are unsure if they want more children, and are currently not using contraception.

Women with unmet need for limiting were delineated as: pregnant women who did not want current pregnancy at all, postpartum amenorrheic women (for less than 24 months) not using contraception who did not want last birth at all, or women who want no more children, and are currently not using contraception.

Infecund women were excluded from the definition of unmet need for spacing and limiting, and they were identified as: married more than 5 years ago, never used contraception, and did not have a child in those years, declared infecund medically, menopause or hysterectomy is the reason for not using contraception, not postpartum amenorrheic and have not had a period in the last 6 months, have had menopause or hysterectomy since last period, never menstruated on time since last period unless they had a birth in the last 5 years, or time since last birth is >  = 60 months, and last period was before the birth.

After calculating the number of women having unmet need for spacing and limiting, the prevalence of each (%) was calculated as:

$$\frac{Number\;of\;women\;with\;unmet\;need\;for\;spacing/limiting\;(ages\;15-49\;years)}{Sample\;population\;of\;women\;ages\;15-49\;years}\times100$$

### Constructing Comparable State Estimates

Geographies of states and UTs in India have changed between 1993 and 2021. Currently, there are 28 states and 8 UTs in India, however in 1993 there were 25 states and 7 UTs [14, 25]. To provide comparable estimates, the usual approach is to combine states of the most recent state-geometry that formed the parent state in the older state-geometry. For example, Andhra Pradesh and Telangana were one state in 1993, thus the conventional approach would be to find the average of estimates from both states and represent them as one. However, this approach has its pitfalls as representing recent data on older state-geometry does not lead to accurate current estimates and is not meaningful for state-level policy formation. By using the methodology described by Subramanian et al. [14], we assigned surveyed districts from previous rounds of the NFHS to their current geometries and calculated state/UT estimates.

### Demographic and Socioeconomic Correlates

We determined the prevalence of total unmet need across demographic and socioeconomic characteristics in 2021. These included age in 5–year groups (15–19/20–24/25–29/30–34/35–39/40–44/45–49 years), religion (Hindu/Muslim/Christian/Other), caste (Scheduled Caste/Scheduled Tribe/Other Backward Class/Other), place of residence (rural/urban), education level (no education/primary/secondary/higher education), and an asset-based categorization of household wealth presented as quintiles (lowest 20%, 20% to 40%, 40% to 60%, 60% to 80%, and highest 20%).

### Analysis

For each round of the NFHS, trends over time for all-India and states/UTs were estimated by calculating the prevalence of unmet need for spacing, limiting, total unmet need, and 95% Confidence Interval. We used the survey weights to account for the multi-stage stratified cluster sampling design. We calculated the Standardized Absolute Change (SAC) to quantify change (in percentage points) in prevalence of total unmet need. This was determined by: $S{\text{AC}}=\frac{{P}_{t}-{P}_{x}}{t-x}$; where ${P}_{t}$ is the total unmet need prevalence in the current year of consideration, ${P}_{x}$ is the total unmet need prevalence in a previous year of consideration, and $t-x$ is the time difference in years. A negative SAC value indicates a decline in the total unmet need, while a positive SAC value represents an increase in unmet need depicting a worsening change, as it implies that total unmet need is moving further away from the target of zero unmet need by 2030.

We used box plots to graphically represent variability in total unmet need in states/UTs over time. We also used a scatterplot to assess whether the magnitude of change in total unmet need from 1993 and 2021 is correlated with total unmet need in 1993 (considered as baseline).

We estimated the absolute burden *i.e.,* current headcount of women with total unmet need for family planning for all-India and states/UTs in 2021 based on Census of India Population Projections for 2021 [26]. The method provided by Integrated Public Use Microdata Series (IPUMS) [27] was utilized assuming a total of 362,865,000 women in India in 2021. This approach has been validated in previously published work [14].

We assessed which states/UTs are on track to meet the ICPD + 25 target of zero unmet need by 2030. The annual absolute change (AAC) in total unmet need was calculated for each state/UT between 2016 and 2021 using the equation:

1 $$AAC = \frac{{P}_{t} - {P}_{x}}{5}$$

where ${P}_{t}$ is the total unmet need prevalence in 2021, and ${P}_{x}$ is the prevalence in 2016. Next, the required annual change (RAC) needed to achieve the target was computed by:

2 $$RAC= \frac{{P}_{t}-{P}_{x}}{14}$$

where ${P}_{t}$ is target total unmet need prevalence in 2030 (0%), and ${P}_{x}$ is prevalence in 2016. While calculating the RAC, it is assumed that the trend between 2016 and 2021 will prevail until 2030, and the progress rate is linear. If the AAC is less than the RAC, the state/UT is on target to meet its 2030 target as the actual rate of change is faster than the required rate of change. Lastly, the predicted year when each state/UT will reach its goal was estimated using:

3 $$Y=\left[\frac{{P}_{t}-{P}_{2021}}{AAC}\right]-9$$

where Y is the number of years after 2030, ${P}_{t}$ is target total unmet need prevalence in 2030 (0%), and ${P}_{2021}$ is the prevalence in 2021. The methodology and use of Eqs. 1, 2 and 3 has been validated in previously published work [28].

The software R [29], Microsoft Power BI [30], and Microsoft Excel [31] were used for computations and visualizations.

### Ethics Statement

Data from all rounds of the NFHS was collected with informed consent of survey participants. Content of all questionnaires was approved by the International Institute for Population Studies Institutional Review Board and the ICF Institutional Review Board [32]. This study does not meet the regulatory definition of human participant research, as defined by the Harvard Longwood Campus Institutional Review Board (IRB) and is exempt from a full institutional review.

## Results

### Sample Characteristics

The number of currently married/in union women of ages 15–49 years varied from 84,289 in 1993 to 512,408 in 2021. The 1993 survey had the largest number of missing responses to unmet need-related questions with 1,183 missing responses, which accounted for 1·4% of all responses that year. The final analytic sample for each survey round is represented in Table 1.

### Patterns of Change in Unmet Need Prevalence

**Fig. 1:**
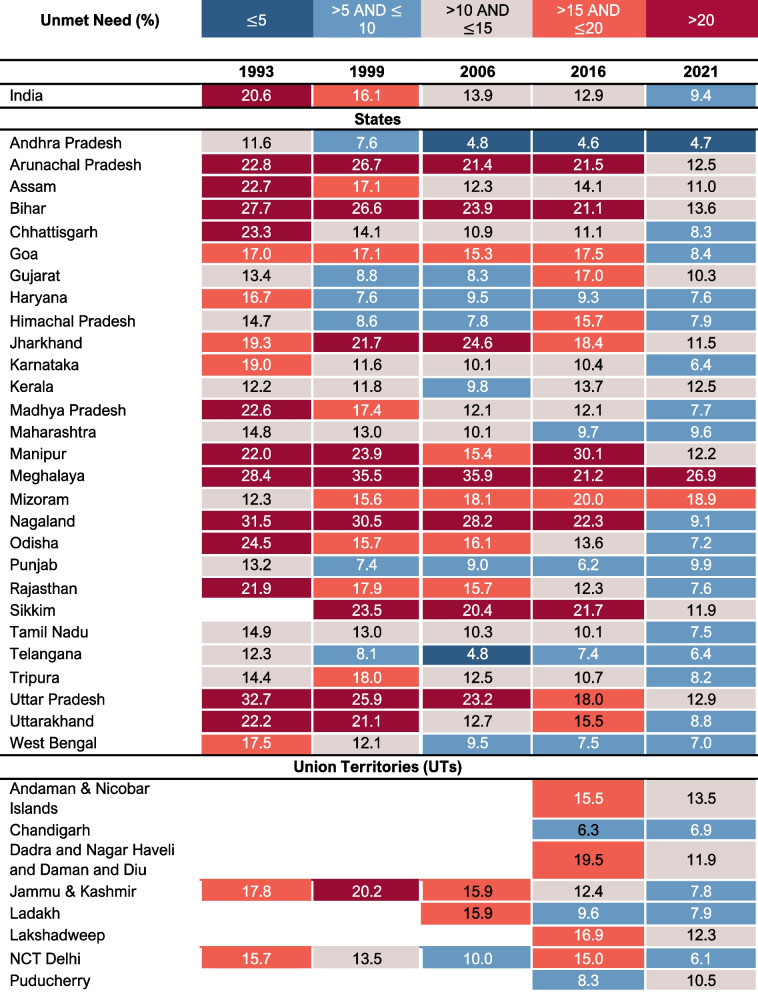
Prevalence of Total Unmet Need for India and 36 States/Union Territories, 1993–2021

**Fig. 2:**
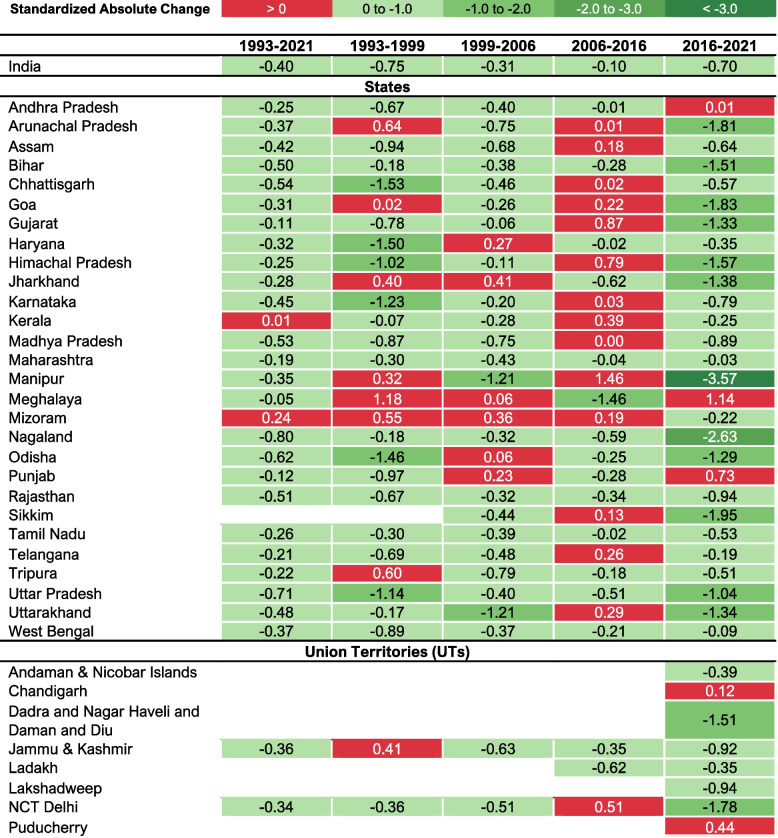
Standardized Absolute Change (Percentage Points) for prevalence of Total Unmet Need

All–India total unmet need prevalence decreased from 20·6% (95% CI: 20·1–21·2%) in 1993, to 9·4% (95% CI: 9·3–9·6%) in 2021 (Table 2 and Fig. 1), representing an absolute annual change of –0·40 percentage points (Fig. 2). The largest decline in total unmet need prevalence occurred between 1993 and 1999, with an annual absolute reduction of 0·75 percentage points.

**Table 2:** Prevalence of Unmet Need (%) and 95% Confidence Interval (CI), 1993–2021

**Survey Year**	**Number of Women (n)**	**Unmet Need (Total) (%)**	**95% CI**	**Number of Women (n)**	**Unmet Need (Spacing) (%)**	**95% CI**	**Number of Women (n)**	**Unmet Need (Limiting) (%)**	**95% CI**
1993	17,120	20.6	[20.1–21.2]	10,281	12.4	[12.0–12.8]	6,839	8.2	[7.9–8.6]
1999	13,634	16.1	[15.7–16.6]	7,011	8.3	[8.0–8.6]	6,623	7.8	[7.5–8.1]
2006	12,950	13.9	[13.5–14.4]	5,668	6.1	[5.8–6.3]	7,282	7.8	[7.5–8.1]
2016	65,750	12.9	[12.7–13.0]	28,883	5.6	[5.5–5.7]	36,867	7.2	[7.1–7.3]
2021	49,125	9.4	[9.3–9.6]	21,087	4.0	[4.0–4.2]	28,037	5.4	[5.3–5.5]

All–India prevalence of unmet need for spacing reduced from 12·4% (95% CI: 10·0– 12·8%) in 1993, to 4·0% (95% CI: 4·0–4·2%) in 2021. Prevalence of unmet need for limiting declined from 8·2% (95% CI: 7·9–8·6%) in 1993, to 5·4% (95% CI: 5·3–5·5%) in 2021 (Table 2).

Assessing the patterns in change of total unmet need prevalence across states/UTs from 1993 to 2021, the largest standardized absolute changes representing worsening prevalence as indicated by positive values, were observed for Kerala (0·01%) and Mizoram (0·24%) (Fig. 2). In the most recent period (2016–2021), Manipur (–3·57%) and Nagaland (–2·63%) had the largest negative standardized absolute change, thus had the greatest decrease in total unmet need prevalence.

Overall, the number of states with prevalence of total unmet need greater than 20% declined in 2021 as compared to 1993, with 12 states in 1993 as opposed to one state in 2021 (Fig. 1 and Additional File 1 Table S1). In 1993, Uttar Pradesh (32·7%, 95% CI: 31·4–34·1%) had the highest prevalence, followed by Nagaland (31·5%, 95% CI: 27·7–35·3%) and Meghalaya (28·4%, 95% CI: 24·7–32·1%). By 2021, the prevalence decreased significantly in Uttar Pradesh (12·9%, 95% CI: 12·5–13·2%) and Nagaland (9·1%, 95% CI: 7·8–10·4%). However, Meghalaya (26·9%, 95% CI: 25·3–28·6%) did not see a large decrease and it was the only state with prevalence greater than 20%.

The number of states with prevalence of unmet need for spacing greater than 15% decreased from seven states in 1993 to one state in 2021 (Additional File 1 Table S2). Meghalaya was the state with the highest prevalence from 1993 (24·1%, 95% CI: 20·8–27·5%) to 2021 (18·3%, 95% CI: 16·7–19·9%). In 1993, Uttar Pradesh (19·1%, 95% CI: 18·0–20·1%) had the second-highest prevalence and it saw a significant decrease by 2021 (4·8%, 95% CI: 4·6–5·0%).

Similarly, the number of states with prevalence of unmet need for limiting greater than 10%, declined from five states in 1993 to zero states in 2021 (Additional File 1 Table S3). In 1993, Nagaland (14·4%, 95% CI: 11·8–16·9%) had the highest prevalence, and it saw a notable decline by 2021 (4·7%, 95% CI: 3·8–5·6%). Meghalaya had a relatively low prevalence in 1993 (4·2%, 95% CI: 3·0–5·4%), however it had the greatest prevalence in 2021 (8·6%, 95% CI: 7·6–9·7%).

**Fig. 3:**
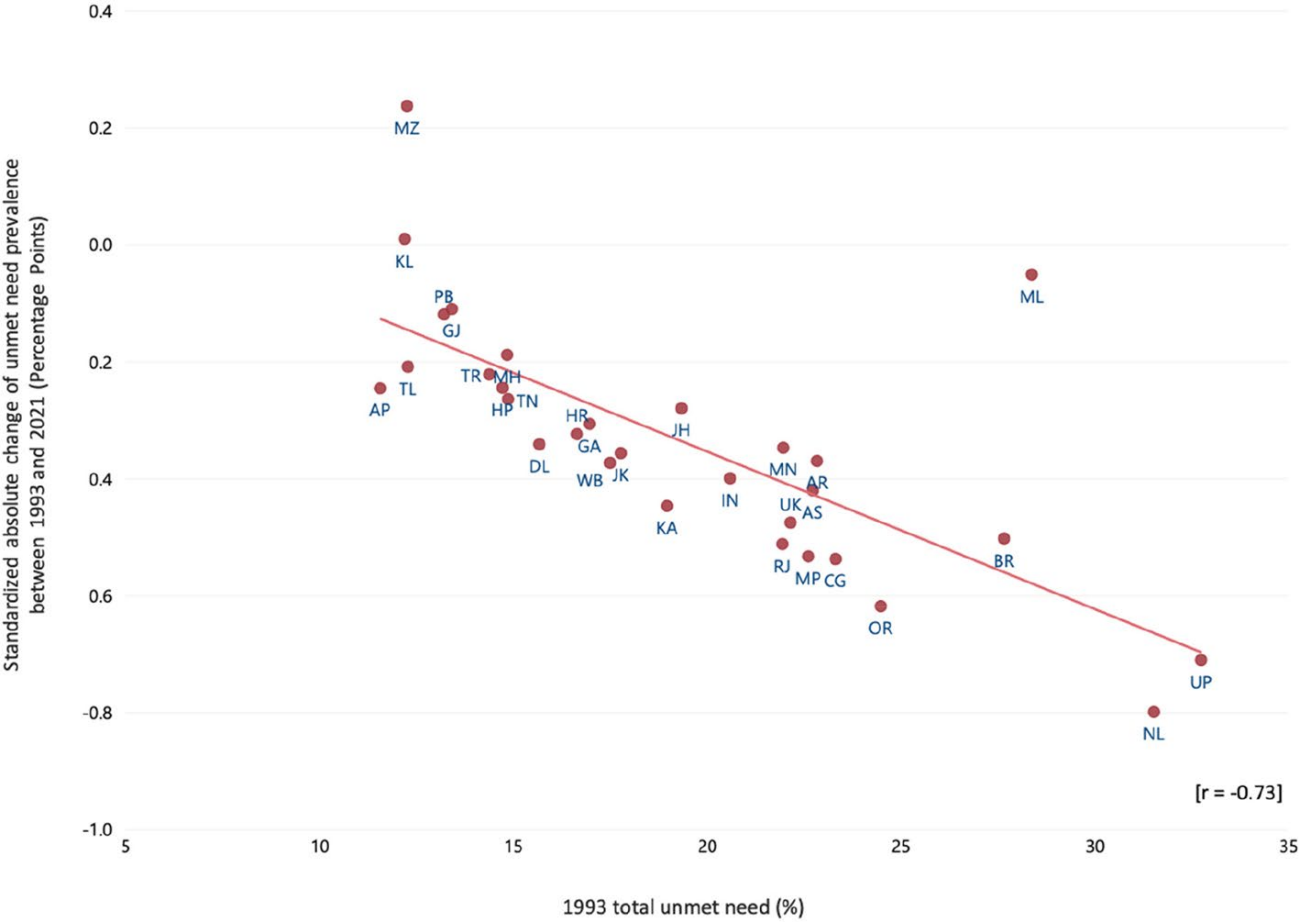
Relationship between 1993 Total Unmet Need Prevalence and Standardized Absolute Change for 1993–2021. Note: AN: Andaman & Nicobar, AP: Andhra Pradesh, AR: Arunachal Pradesh, AS: Assam, BR: Bihar, CH: Chandigarh, CG: Chhattisgarh, DH: Dadra and Nagar Haveli and Daman and Diu, GA: Goa, GJ: Gujarat, HR: Haryana, HP: Himachal Pradesh, JK: Jammu & Kashmir, JH: Jharkhand, KA: Karnataka, KL: Kerala, LK: Ladakh, LD: Lakshadweep, MP: Madhya Pradesh, MH: Maharashtra, MN: Manipur, ML: Meghalaya, MZ: Mizoram, DL:NCT Delhi, NL: Nagaland, OR: Odisha, PY: Puducherry, PB: Punjab, RJ: Rajasthan, SK: Sikkim, TN: Tamil Nadu, TL: Telangana, TR: Tripura, UP: Uttar Pradesh, UK: Uttarakhand, WB: West Bengal

It was discerned that total unmet need prevalence in 1993 (baseline) was inversely associated with the standardized absolute change between 1993 and 2021 (*r* = –0·73), i.e. states with a higher prevalence in 1993 experienced a greater decline on average (Fig. 3).

### Changes in the Geographic Distribution of Unmet Need Prevalence

**Fig. 4:**
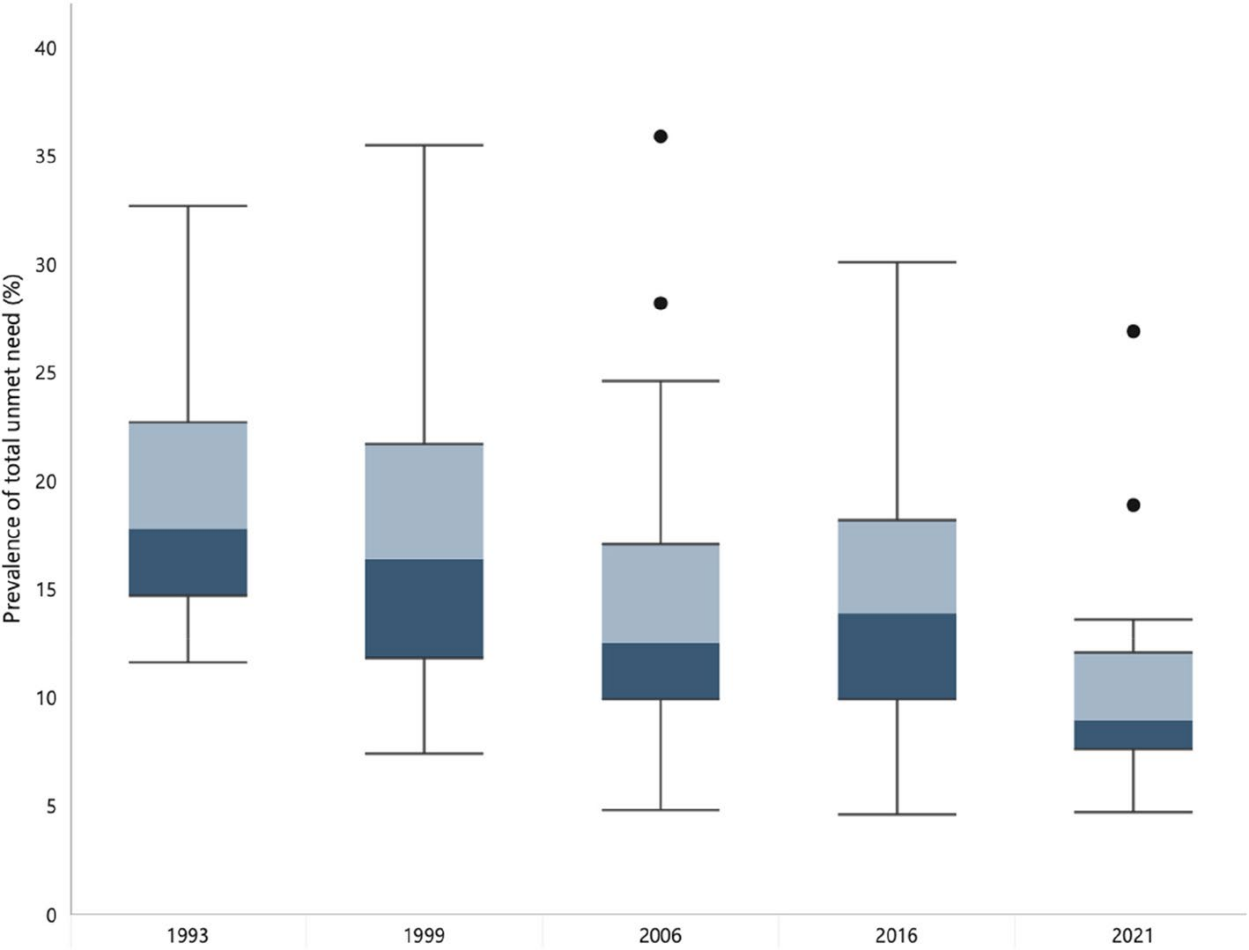
Summary distribution of state/union territory-level Total Unmet Need for family planning, 1993–2021. Note: Box-and-whisker plots show the variability of a data set using lowest and highest values, and quartiles of the data. The upper and lower whiskers represent minimum and maximum values respectively. The upper outline of the box depicts 75th percentile and the lower outline represents the 25th percentile. The line within the box shows the median (i.e., 50th percentile)

Inequalities in prevalence among states and UTs, measured by interquartile range (IQR), which is a measure of statistical variability, significantly decreased from 1993 to 2021, with a sharp decline after 2016 (Fig. 4). The IQR decreased from 8·0% (25th percentile: 14·7%, 75th percentile: 22·7%) in 1993 to 4·5% (25th percentile: 7·6%, 75th percentile: 12·1%) in 2021 (Additional File 1 Table S4).

In 2021, total unmet need was concentrated in the north and north east with Meghalaya (26·9%, 95% CI: 25·3–28·6%) and Mizoram (18·9%, 95% CI: 17·2–20·6%) having the highest total unmet need prevalence, followed by the northern states of Bihar (13·6%, 95% CI: 13·1–14·1%) and Uttar Pradesh (12·9%, 95% CI: 12·5–13·2%) (Additional File 1 Table S1). Substantially lower prevalence were noted in the southern states of Andhra Pradesh (4·7%, 95% CI: 4·2–5·2%), Karnataka (6·4%, 95% CI: 6·0–6·9%), and Telangana (6·4%, 95% CI: 6·0–6·9%), and the northern UT of NCT Delhi (6·1%, 95% CI: 5·4–6·8%). A similar geographic distribution was observed in 1993.

Unmet need for spacing was similar amongst northern and southern states in 2021. However, higher prevalence was observed in north-eastern states, and Mizoram (12·9%, 95% CI: 11·2–14·5%) and Meghalaya (18·3%, 95% CI: 16·7–19·9%) had the highest values (Additional File 1 Table S2). This trend was also observed in 1993, with Arunachal Pradesh (15·4%, 95% CI: 12·6–18·3%), Nagaland (17·2%, 95% CI: 14·0–20·3%), and Meghalaya (24·1%, 95% CI: 20·8–27·5%) having some of the highest values.

In 2021, the unmet need for limiting was substantially lower in most southern states as compared to northern states, and the southern state of Andhra Pradesh had the lowest prevalence (2·0%, 95% CI: 1·7–2·4%) (Additional File 1 Table S3). Similarly, in 1993, northern states tended to have higher prevalence than other geographical areas. The states of Bihar (10·6%, 95% CI: 9·5–11·8%), Uttarakhand (11·4%, 95% CI: 8·3–14·4%), and Uttar Pradesh (13·7%, 95% CI: 12·8–14·6%) had higher prevalence values than the national estimate in 1993.

It was estimated that 17 states/UTs are on track to meet the ICPD + 25 target of zero unmet need by 2030, while 19 will not, based on their current trajectories (Additional File 1 Table S5). All southern states/UTs except Karnataka are off-target, and Andhra Pradesh is expected to never reach the target at its current rate as the AAC from 2016–2021 in total unmet need prevalence (0·01%) was lower than the RAC (–0·3%). The negative value indicates that a decline of 0·3% per year is required, however an increase of 0·01% per year from 2016–2021 was observed. Meghalaya, Punjab, Chandigarh, and Puducherry are also expected to never reach the target due to having lower AAC than RAC. Maharashtra, Mizoram and West Bengal are expected to reach the target after 2100, if the current AAC persists.

### Estimated Headcount of Total Unmet Need Prevalence

We estimated that 24,194,428 women had an unmet need for family planning in 2021. The headcount varied from 5,140,642 in Uttar Pradesh to 1,793 in Lakshadweep (Table 3). Nine of these states accounted for approximately 75% of the population with unmet need. These were Uttar Pradesh (21·25%), Bihar (12·78%), Maharashtra (9·65%), West Bengal (6·72%), Gujarat (5·11%), Rajasthan (4·88%), Madhya Pradesh (4·77%), Tamil Nadu (4·74%), and Karnataka (3·58%). Of the UTs, NCT Delhi was the largest contributor with 220,630 women (0·91%).

**Table 3:** Estimated headcount of women with Unmet Need for India and 36 States/Union Territories, 2021

	**Total Unmet Need**	**Percentage Distribution**
India	24,194,428	100
**States/Union Territories**		
Uttar Pradesh	5,140,642	21.25
Bihar	3,093,138	12.78
Maharashtra	2,335,020	9.65
West Bengal	1,626,715	6.72
Gujarat	1,236,773	5.11
Rajasthan	1,181,798	4.88
Madhya Pradesh	1,153,615	4.77
Tamil Nadu	1,146,885	4.74
Karnataka	865,203	3.58
Kerala	826,378	3.42
Jharkhand	773,040	3.20
Assam	757,727	3.13
Odisha	615,106	2.54
Punjab	537,114	2.22
Andhra Pradesh	500,544	2.07
Chhattisgarh	467,139	1.93
Telangana	446,552	1.85
Haryana	378,162	1.56
NCT Delhi	220,630	0.91
Uttarakhand	181,843	0.75
Jammu & Kashmir	153,825	0.64
Meghalaya	147,219	0.61
Himachal Pradesh	110,108	0.46
Tripura	71,228	0.29
Manipur	49,599	0.21
Mizoram	30,161	0.12
Goa	28,092	0.12
Arunachal Pradesh	25,175	0.10
Puducherry	23,760	0.10
Nagaland	19,699	0.08
Chandigarh	13,486	0.06
Sikkim	12,848	0.05
Dadra and Nagar Haveli and Daman and Diu	11,274	0.05
Andaman & Nicobar Islands	9,417	0.04
Ladakh	2,720	0.01
Lakshadweep	1,793	0.01

### Correlates of Total Unmet Need Prevalence

We found that all-India total unmet need prevalence in 2021 was the greatest among women of ages 15–19 (17·8%, 95% CI: 17·0–18·6%), followed by women of ages 20–24 (17·3%, 95% CI: 16·9–17·7%). When patterned by religion and caste, unmet need was highest among Muslim women (11·8%, 95% CI: 11·3–12·3%), and women from Other Backward Class households (9·6%, 95% CI: 9·4–9·8%). It was found that larger proportion of women living in rural areas experience unmet need (9·9%, 95% CI: 9·7–10·0%), than those living in urban areas (8·4%, 95% CI: 8·2–8·7%). Unmet need prevalence increased with level of education attained and 12·6% (95% CI: 12·1–13·1%) of women with higher education had an unmet need for family planning. Patterning by wealth quintile depicted that the prevalence in the poorest group was approximately three percentage points higher than the richest group (Table 4 and Additional File 1 Table S6).

**Table 4:** Prevalence of Total Unmet Need (%) by Demographic and Socioeconomic characteristics, 2021

**Factor**		**Number of Women (n)**	**Unmet Need 2021 (Total) (%)**	**95% CI**
**Age (grouped by 5 years)**	15–19	2,742	17.8	[17.0–18.6]
	20–24	12,403	17.3	[16.9–17.7]
	25–29	13,497	13.2	[12.9–13.5]
	30–34	8,514	9.1	[8.8–9.4]
	35–39	5,743	6.3	[6.1–6.6]
	40–44	3,698	5.0	[4.8–5.2]
	45–49	2,528	3.4	[3.2–3.6]
**Religion**	Hindu	38,539	9.0	[8.9–9.2]
	Muslim	8,097	11.8	[11.3–12.3]
	Christian	1,180	10.4	[9.6–11.1]
	Other	1,310	9.2	[8.5–9.9]
**Caste**	Scheduled Caste	10,340	9.2	[8.9–9.4]
	Scheduled Tribe	4,423	9.2	[8.9–9.6]
	Other Backward Class	21,516	9.6	[9.4–9.8]
	Other	10,416	9.4	[9.1–9.8]
**Place of residence**	Rural	35,355	9.9	[9.7–10.0]
	Urban	13,770	8.4	[8.2–8.7]
**Education Level**	No education	10,425	7.3	[7.1–7.5]
	Primary	5,667	7.9	[7.6–8.1]
	Secondary	24,568	10.3	[10.1–10.5]
	Higher	8,466	12.6	[12.1–13.1]
**Wealth Quintile**	Poorest	11,135	11.4	[11.1–11.7]
	Poor	10,097	9.7	[9.4–9.9]
	Middle	9,197	8.6	[8.4–8.9]
	Rich	9,732	9.0	[8.7–9.3]
	Richest	8,964	8.6	[8.3–8.9]

## Discussion

This study has five salient findings. First, India has made significant progress in eliminating unmet need for family planning over the last 30 years. The prevalence of total unmet need has decreased by 11·2 percentage points since 1993. However, the change in unmet need for spacing and unmet need for limiting remained fairly constant between 2006 and 2016. Second, inequalities in total unmet need prevalence across states/UTs significantly decreased between 1993 and 2021 by 3·5 percentage points. But, a number of states/UTs had prevalence values greater than the national estimate in 2021 and no state/UT reached the ICPD + 25 target of zero unmet need for family planning. Geographical disparities were observed in total unmet need prevalence for 2021, with some southern states having the lowest values. Furthermore, unmet need for limiting was substantially lower amongst southern states in 2021, while unmet need for spacing was similar among northern and southern states. This may be explained by the high rates of female sterilization that are present in south India. More than 80% of women in south India have been shown to rely on female sterilization over the past two decades [33]. Third, states/UTs that had a higher total unmet need prevalence in 1993, experienced a greater standardized absolute change between 1993 and 2021. Fourth, India currently has a substantial headcount of women with an unmet need for family planning. Uttar Pradesh, Bihar, Maharashtra and West Bengal account for half of this population. The headcount points to different geographies that prevalence alone does not prioritize. While Meghalaya had the highest prevalence of total unmet need in 2021, it had the 15th lowest headcount across all states/UTs. Thus, along with the prevalence, the headcount must be considered to determine states/UTs that need to prioritized as part of the National Family Planning Programme. Fifth, there are considerable differences in total unmet need prevalence across demographic and socioeconomic characteristics. It was observed that younger women and those in the lowest quintiles of wealth had relatively higher prevalence of total unmet need in 2021.

The following limitations of the study must be considered while interpreting the results. First, household surveys may not include all currently married/in union women as certain rounds of the NFHS did not cover all the states and UTs of India, which may have an impact on the estimates for those rounds [18]. For example, the first round of the NFHS (1993) did not cover Sikkim and the UTs except Jammu & Kashmir and NCT Delhi [34]. Data from all states/UTs was only available from 2016–2021. Second, the currently married/in union population has different definitions across all surveys. Third, an integral question for determining unmet need pertaining to the desire for more children was only asked to currently married or ever married women in the first three rounds of the survey. Hence, trends in unmet need could not be ascertained for unmarried women. From a policy perspective, it is valuable to find the prevalence in this population group, even though they are not included in the definition of unmet need proposed by the United Nations [35]. Unmarried women may be sexually active and experience unmet need, which should be eradicated to achieve the ICPD + 25 goal of zero unmet need.

Fourth, India’s most recent census was conducted in 2011, due to delays as a result of the COVID-19 pandemic [36]. There have been reports of considerable uncertainty about the current demographics in India, due to outdated data [37]. This may affect the headcount estimates as the total number of women in 2021 was derived from population projections from Census 2011. Fifth, state boundaries in India have changed over the years and approximations have been provided by constructing comparable state estimates [14]. For 1993 and 1999, estimates for Andhra Pradesh, Bihar, Chhattisgarh, Jharkhand, Madhya Pradesh, Telangana, Uttar Pradesh, Uttarakhand, and Jammu and Kashmir are approximations. For 2006, prevalence values for Andhra Pradesh, Telangana, Jammu and Kashmir, and Ladakh, and for 2016, estimates for Jammu and Kashmir, and Ladakh are approximations. Lastly, there is a substantial amount of debate in literature about the current definition of unmet need for family planning as having unmet need does not take into account a woman’s stated desire for contraception. Efforts to reduce unmet need must ensure that coercive measures are not taken to increase family planning use [38]; this is especially important in a context like India where there is heavy reliance on female sterilization.

Despite these limitations, this study provides useful insights for policy deliberation. Prior studies have shown that unmet need for family planning is greatest among women of ages 15–19 years and those with the lowest education levels and belonging to the poorest households [12, 39]. This study corroborates the claim that women of ages 15–19 years and those belonging to the poorest wealth quintile have the highest prevalence of unmet need, however, in 2021, women with the highest level of education had greater unmet need than women with lower levels of education. As per a previous study amongst married women of ages 15–19 years in India, child marriage facilitates limited knowledge, and autonomy for contraceptive use, thus decreasing the likelihood of its use in this population [40]. Although this study reveals that the women with the highest level of education had greater unmet need than women in other educational attainment categories, it must be noted that the unmet need for spacing increased with education level, while unmet need for limiting remained fairly constant (Additional File 1 Table S6). This suggests that women with more education are more interested in spacing than limiting, however, limiting methods are most common in India, especially female sterilization [41]. Thus, there may be a need for improving programs for spacing methods to better meet women's reproductive intentions as preferences change. Multiple initiatives have been launched under the National Family Planning Programme including Mission Parivar Vikas, launched in 2016, to increase access to contraceptives and family planning services in seven states with high total fertility rates [42]. These states were Uttar Pradesh, Bihar, Rajasthan, Madhya Pradesh, Chhattisgarh, Jharkhand and Assam and all states showed a considerable decline in total unmet need prevalence in 2021, as estimated in this study. However, Mission Parivar Vikas did not include the states of Maharashtra and West Bengal, which were in the top four states with high headcount of women with unmet need in 2021. Under the National Family Planning Programme, emphasis has been placed on increasing access to spacing methods, specifically the use of Intra-Uterine Contraceptive Devices (IUCDs) [43]. Furthermore, an injectable hormonal contraceptive and a contraceptive pill under the Antara and Chhaya programs respectively, were launched in ten states with high headcount of women with unmet need such as Uttar Pradesh, and Maharashtra [44]. Despite these policies, the use of spacing methods has been fairly stagnant and women in India largely depend on limiting methods, especially female sterilization [41]. Furthermore, a recent study indicated that rates of male contraception in India are low, and the burden of family planning falls on women, with 40.2% of men believing that women are responsible for avoiding pregnancy [45]. This indicates that there may also be a need to implement programs to increase male participation in family planning [45].

These findings lend strength to the urgency of addressing the prevalence of unmet need for family planning in India. The framework of the National Family Planning Programme should be re-examined with a focus on states with high headcount of women with unmet need for family planning. Policies should be formulated specific to population groups with relatively high unmet need, such as women of ages 15–19 and women belonging to the poorest wealth quintile. Bringing precision to India’s existing family planning policies should be urgently considered if India aims to achieve zero unmet need in all states/UTs by 2030.

### Supplementary Information

**Supplementary Material 1.**

## Data Availability

The study is based on publicly available data and can be accessed from https://dhsprogram.com/data/available-datasets.cfm.
