# Supplementary material for: Trends in prevalence of unmet need for family planning in India: patterns of change across 36 States and Union Territories, 1993–2021
Source: Reprod Health. 2024 Apr 9;21:48. doi: 10.1186/s12978-024-01781-6 (PMC11003006; doi:10.1186/s12978-024-01781-6)
Supplement: Supplementary file 1 — Supplementary Material 1. [file 12978_2024_1781_MOESM1_ESM.docx]

**Additional File 1**

**Trends in prevalence of unmet need for family planning in India: Patterns of change across 36 States and Union Territories, 1993-2021**

Table of Contents

[Supplementary Table 1: Prevalence of Total Unmet Need (%) and 95% Confidence Interval (CI) (ages 15-49 years) for India and 36 States/Union Territories, 1993-2021 2](#_Toc159199012)

[Supplementary Table 2: Prevalence of Unmet Need for Spacing (%) and 95% Confidence Interval (CI) (ages 15-49 years) for India and 36 States/Union Territories, 1993-2021 3](#_Toc159199013)

[Supplementary Table 3: Prevalence of Unmet Need for Limiting (%) and 95% Confidence Interval (CI) (ages 15-49 years) for India and 36 States/Union Territories, 1993-2021 4](#_Toc159199014)

[Supplementary Table 4: Standard Deviation (SD) and Interquartile Range (IQR) of Total Unmet Need prevalence in States/Union Territories of India, 1993-2021 5](#_Toc159199015)

[Supplementary Table 5: Progress towards ICPD+25 target by State/UT 6](#_Toc159199016)

[Supplementary Table 6: Prevalence of Unmet Need for Spacing (%) and Unmet Need for Limiting (%) by Demographic and Socioeconomic characteristics, 2021 7](#_Toc159199017)

# **Supplementary Table 1: Prevalence of Total Unmet Need (%) and 95% Confidence Interval (CI) (ages 15-49 years) for India and 36 States/Union Territories, 1993-2021**


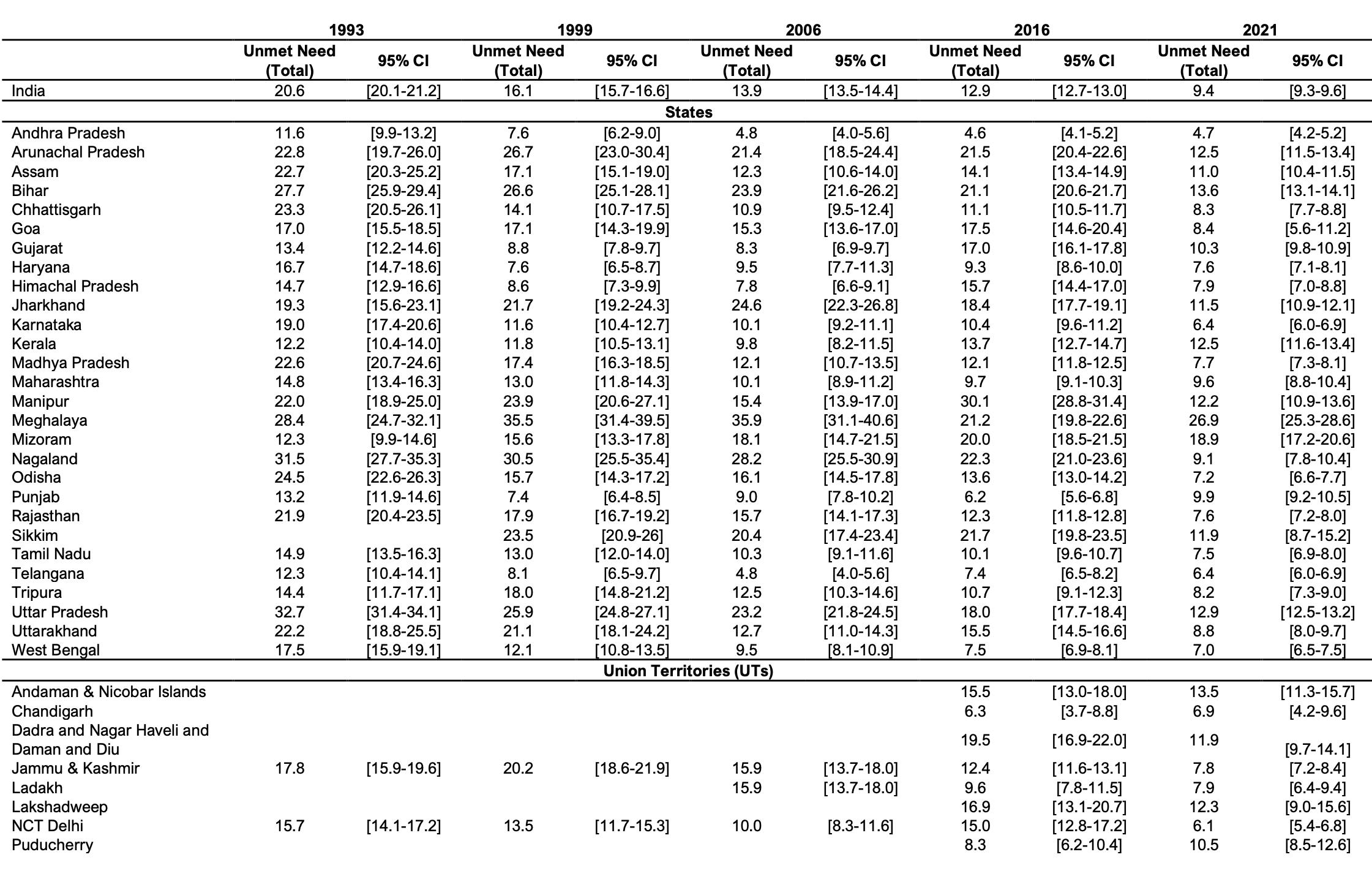


# **Supplementary Table 2: Prevalence of Unmet Need for Spacing (%) and 95% Confidence Interval (CI) (ages 15-49 years) for India and 36 States/Union Territories, 1993-2021**


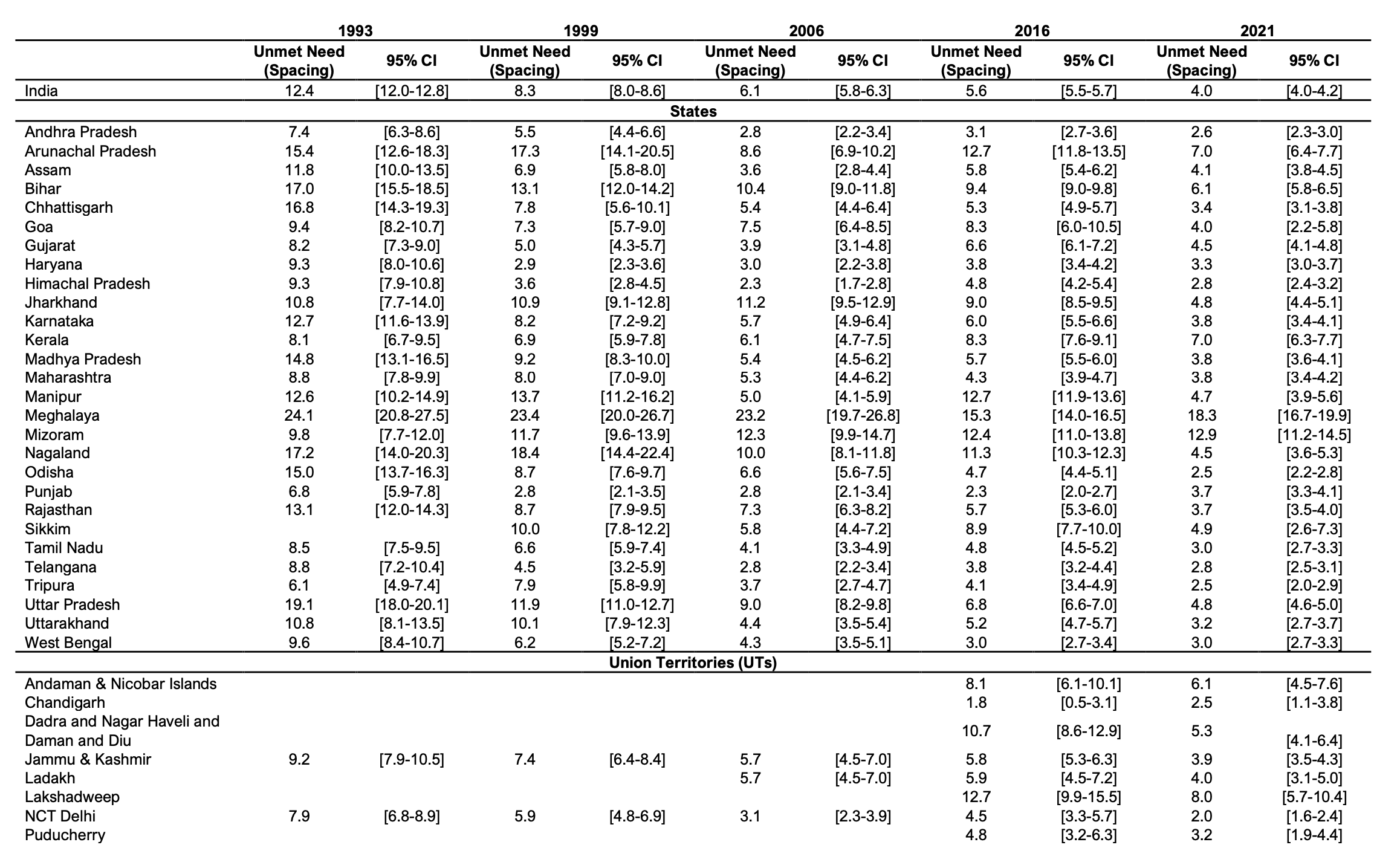


# **Supplementary Table 3: Prevalence of Unmet Need for Limiting (%) and 95% Confidence Interval (CI) (ages 15-49 years) for India and 36 States/Union Territories, 1993-2021**


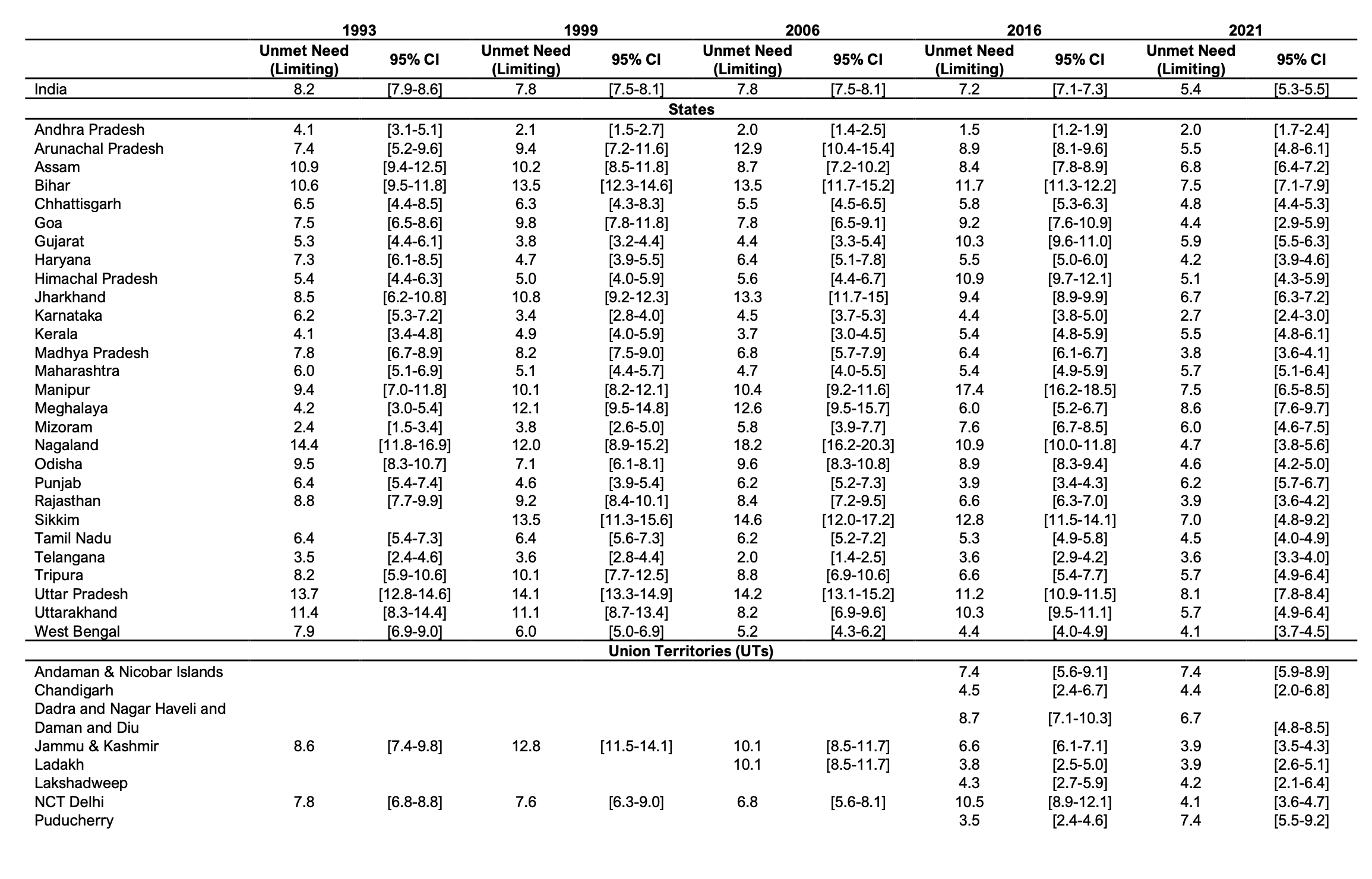


# **Supplementary Table 4: Standard Deviation (SD) and Interquartile Range (IQR) of Total Unmet Need prevalence in States/Union Territories of India, 1993-2021**

| **Survey Year** | **Total unmet need** | |
| --- | --- | --- |
|  | **SD** | **IQR** |
| 1993 | 5.9 | 8.0 |
| 1999 | 7.3 | 9.9 |
| 2006 | 7.1 | 7.2 |
| 2016 | 5.7 | 8.3 |
| 2021 | 4.1 | 4.5 |

**Note**: Values are rounded to 1 decimal place.

# **Supplementary Table 5: Progress towards ICPD+25 target by State/UT**


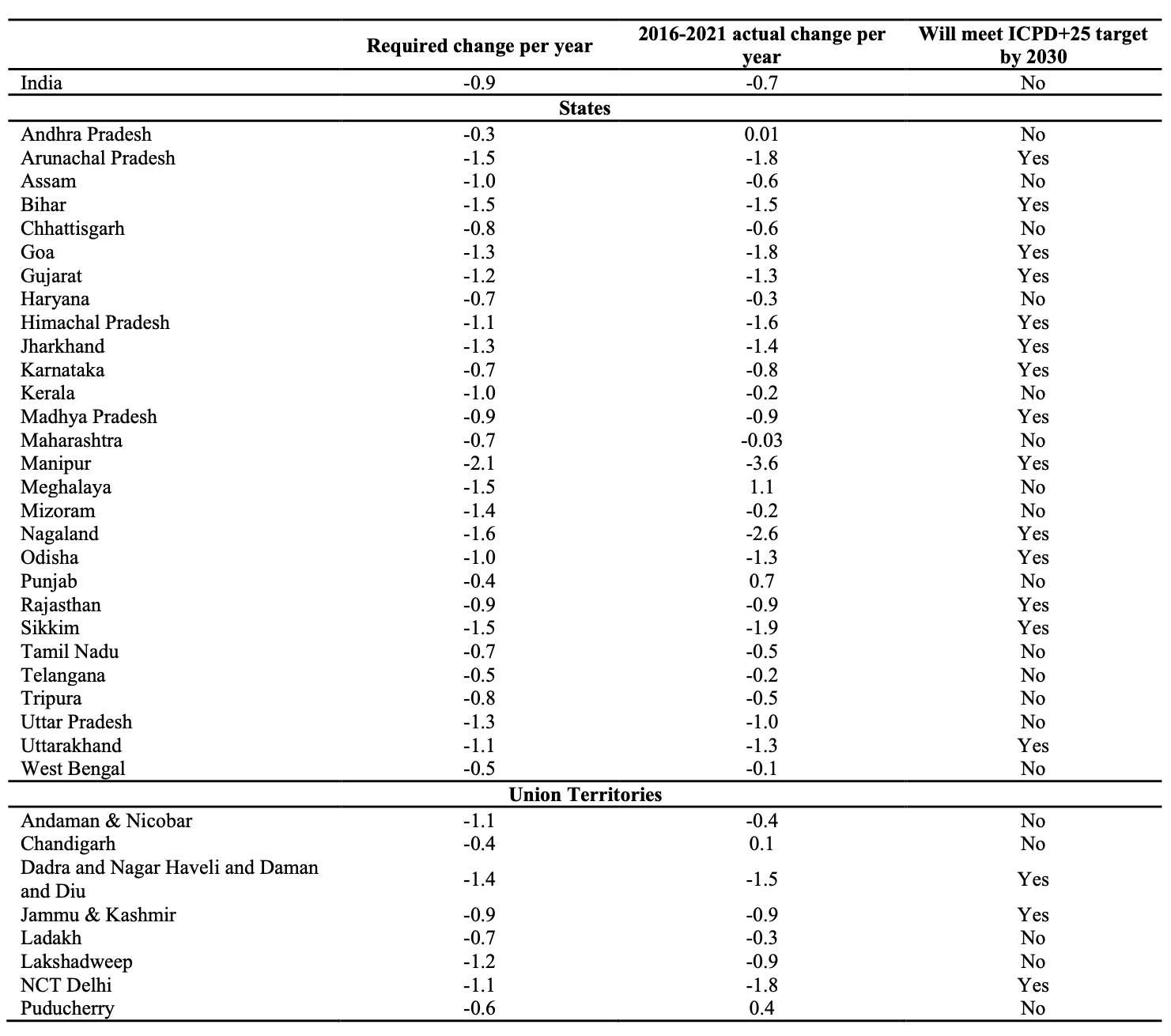


# **Supplementary Table 6: Prevalence of Unmet Need for Spacing (%) and Unmet Need for Limiting (%) by Demographic and Socioeconomic characteristics, 2021**

|  | | | | |  |  |  |
| --- | --- | --- | --- | --- | --- | --- | --- |
| **Factor** |  | **Number of Women (n)** | **Unmet Need 2021 (Spacing) (%)** | **95% CI** | **Number of Women (n)** | **Unmet Need 2021 (Limiting) (%)** | **95% CI** |
| **Age (grouped by 5 years)** | 15-19 | 2,401 | 15.6 | [14.8-16.3] | 342 | 2.2 | [1.9-2.6] |
|  | 20-24 | 8,899 | 12.4 | [12.1-12.8] | 3,504 | 4.9 | [4.6-5.1] |
|  | 25-29 | 6,318 | 6.2 | [5.9-6.4] | 7,180 | 7.0 | [6.8-7.2] |
|  | 30-34 | 2,347 | 2.5 | [2.4-2.6] | 6,167 | 6.6 | [6.3-6.8] |
|  | 35-39 | 771 | 0.9 | [0.8-0.9] | 4,971 | 5.5 | [5.3-5.7] |
|  | 40-44 | 226 | 0.3 | [0.3-0.4] | 3,472 | 4.7 | [4.5-4.9] |
|  | 45-49 | 126 | 0.2 | [0.1-0.2] | 2,402 | 3.3 | [3.1-3.4] |
|  | | | | | | | |
| **Religion** | Hindu | 16,474 | 3.9 | [3.8-3.9] | 22,063 | 5.2 | [5.0-5.3] |
|  | Muslim | 3,441 | 5.0 | [4.7-5.3] | 4,656 | 6.8 | [6.5-7.1] |
|  | Christian | 630 | 5.5 | [5.0-6.1] | 550 | 4.8 | [4.3-5.3] |
|  | Other | 542 | 3.8 | [3.3-4.3] | 768 | 5.4 | [4.9-5.9] |
|  | | | | | | | |
| **Caste** | Scheduled Caste | 4,474 | 4.0 | [3.8-4.1] | 5,865 | 5.2 | [5.0-5.4] |
|  | Scheduled Tribe | 2,169 | 4.5 | [4.3-4.8] | 2,254 | 4.7 | [4.4-5.0] |
|  | Other Backward Class | 9,397 | 4.2 | [4.1-4.3] | 12,118 | 5.4 | [5.3-5.5] |
|  | Other | 4,076 | 3.7 | [3.5-3.9] | 6,340 | 5.7 | [5.5-6.0] |
|  | | | | | | | |
| **Place of residence** | Rural | 15,289 | 4.3 | [4.2-4.4] | 20,065 | 5.6 | [5.5-5.7] |
|  | Urban | 5,799 | 3.6 | [3.4-3.7] | 7,971 | 4.9 | [4.7-5.1] |
|  | | | | | | | |
| **Education Level** | No education | 2,834 | 2.0 | [1.9-2.1] | 7,591 | 5.3 | [5.2-5.5] |
|  | Primary | 1,857 | 2.6 | [2.4-2.7] | 3,809 | 5.3 | [5.1-5.5] |
|  | Secondary | 11,630 | 4.9 | [4.7-5.0] | 12,938 | 5.4 | [5.3-5.5] |
|  | Higher | 4,768 | 7.1 | [6.8-7.4] | 3,698 | 5.5 | [5.1-5.9] |
|  | | | | | | | |
| **Wealth Quintile** | Poorest | 4,424 | 4.5 | [4.3-4.7] | 6,712 | 6.9 | [6.6-7.1] |
|  | Poor | 4,270 | 4.1 | [3.9-4.3] | 5,827 | 5.6 | [5.4-5.8] |
|  | Middle | 4,137 | 3.9 | [3.7-4.0] | 5,060 | 4.8 | [4.6-4.9] |
|  | Rich | 4,358 | 4.0 | [3.9-4.2] | 5,373 | 5.0 | [4.7-5.2] |
|  | Richest | 3,898 | 3.7 | [3.5-3.9] | 5,066 | 4.9 | [4.6-5.1] |
